# Domain-driven verbal and non-verbal dissociations in cognition and social cognition in Parkinson’s disease

**DOI:** 10.3389/fpsyg.2026.1775416

**Published:** 2026-05-21

**Authors:** S. Bottiroli, M. Cangelosi, S. Lecce, S. Bernini, M. Avenali, C. Tassorelli, T. Vecchi, E. Cavallini

**Affiliations:** 1 Department of Brain and Behavioral Sciences, University of Pavia, Pavia, Italy; 2 IRCCS Mondino Foundation, Pavia, Italy

**Keywords:** cognition, non-verbal components, Parkinson’s disease, theory of mind, verbal components

## Abstract

**Introduction:**

Beyond motor symptoms, Parkinson’s disease (PD) affects cognition and social cognition. This is already evident in cognitively intact PD, but is especially prominent in those with mild cognitive impairment (PD-MCI). The impact of processing modality (verbal vs. non-verbal) remains unclear. We examined differences across verbal and non-verbal components of cognition and Theory of Mind (ToM) in subjects with PD or PD-MCI compared to healthy controls (HC).

**Methods:**

Seventy-seven participants (HC = 26; PD = 28; PD-MCI = 23) completed a battery of neuropsychological tests to assess verbal and non-verbal cognition, verbal and non-verbal ToM. Data were analyzed using non-parametric statistics and multinomial logistic regression models.

**Results:**

Both PD groups showed statistically significant impairments compared to HC, with more pronounced deficits in PD-MCI. Verbal cognition was reduced in both PD groups (HC = 3.55 ± 0.59; PD = 2.76 ± 0.95; PD-MCI = 2.73 ± 0.95; χ^2^ = 27.46, *p* < 0.001, η^2^ = 0.34), while non-verbal cognition was only affected in PD-MCI (HC = 3.54 ± 1.92; PD-MCI = 2.59 ± 1.40; χ^2^ = 16.17, *p* < 0.001, η^2^ = 0.19). Verbal ToM was selectively impaired in PD-MCI (HC = 6.27 ± 1.00; PD-MCI = 4.94 ± 1.95; χ^2^ = 9.07, *p* = 0.01, η^2^ = 0.10), while non-verbal ToM was substantially preserved (*p* > 0.33). The regression analysis confirmed that lower scores in verbal and non-verbal cognition were associated with a higher likelihood of PD-MCI classification.

**Discussion:**

The pattern emerging from our findings is consistent with a framework in which domain-specific vulnerabilities are primary, while modality may influence performance in interaction with task complexity and executive demand. This 2 × 2 framework (domain X modality) refines our understanding of cognitive decline in PD and underscores the need for domain- and modality-sensitive assessments.

## Introduction

Understanding how Parkinson’s disease (PD)—one of the most common neurodegenerative disorders characterized by motor symptoms and involving more than 1% of individuals aged 65 years and older (Balestrino and Schapira, 2020; Bhidayasiri et al., 2024)—affects the mind requires more than cataloging cognitive deficits. PD is a multifaceted disease that deserves careful analysis of how deficits manifest across different functional domains and aspects, with cognitive and social-cognitive dysfunctions increasingly recognized as core features of the disease (Obeso et al., 2017; Maggio et al., 2024). Cognitive deficits, which include impairments in attention, psychomotor speed, episodic memory, executive functioning, and categorical fluency (Elgh et al., 2009), occur up to six times more frequently than in age-matched healthy controls (Aarsland et al., 2011; Wild et al., 2013; Gonzalez-Latapi et al., 2021). Social-cognitive dysfunction is primarily reflected in the ability to infer others’ mental states, such as beliefs, emotions, and intentions, known as Theory of Mind (ToM; Howlin et al., 1999; Bodden et al., 2010; Poletti et al., 2011; Rossetto et al., 2018; Menozzi et al., 2025). All of these impaired domains result in deficits affecting also daily functioning and instrumental activities of daily living (Giannouli and Tsolaki, 2019), underscoring the importance of understanding these processes to mitigate the progression of cognitive decline.

In more detail, it is currently unclear whether these impairments are specific to particular cognitive or social-cognitive domains, whether they are associated with a specific processing modality (verbal versus non-verbal), or whether they instead reflect broader dysfunctions in shared systems, such as executive control. From a neurocognitive perspective, impairments in tasks with higher verbal and executive demands are thought to be primarily related to fronto-striatal dysfunction, a core feature of PD, which critically affects executive control processes. In contrast, deficits in non-verbal and visuospatial tasks have more often been associated with posterior cortical involvement, particularly in parietal regions supporting visuospatial processing (Foley et al., 2019; Kaup et al., 2024). Within this framework, differences between verbal and non-verbal performance may, therefore, reflect not only modality-specific processing, but also the differential contribution of executive control and the involvement of partially distinct neural systems.

Of the various dimensions along which these impairments can be studied (Seyfarth and Cheney, 2015; Rosch, 2024), two stand out as being foundational for human cognition and interaction (Fujita and Fujita, 2022): verbal and non-verbal dimensions. Indeed, these modalities are the two principal channels through which individuals encode, interpret, and express mental states, solve problems, and navigate the social world (Paivio, 2014).

Verbal processing involves language-based operations, such as lexical access, narrative comprehension, and inferential reasoning (Schwering and MacDonald, 2020), while non-verbal processing involves visuo-spatial cognition, visual attention, gesture interpretation, and other non-linguistic cues (Özer and Göksun, 2020). Accordingly, verbal and non-verbal modalities differ not only in representational format, but also in the neural systems they predominantly engage and in the level of executive control required. In particular, verbal tasks more strongly rely on fronto-striatal circuits supporting executive and language-related processes, whereas non-verbal tasks more heavily involve posterior cortical regions, especially parietal areas subserving visuospatial processing (Booth et al., 2007).

Direct comparisons of verbal and non-verbal cognitive performance in PD are scarce (Jaywant et al., 2014), but existing evidence suggests that these processes may be differentially affected (Kemps and Newson, 2006; Foley et al., 2019; D’Antuono et al., 2022). However, many studies combine cognitively normal and mildly impaired patients, limiting the clarity of domain-specific patterns (Jaywant et al., 2014). Regarding the verbal domain in PD, findings remain mixed (Aveni et al., 2023; Novakova et al., 2023). Some studies suggest that higher-order verbal abilities, such as vocabulary and semantic comprehension, remain relatively intact even when cognitive decline is present (Altmann and Troche, 2011; Diaz et al., 2016; Cangelosi et al., 2025). For example, individuals with PD can use combinatory semantic cues effectively to predict upcoming words when processing sentences, which suggests that certain aspects of semantic processing remain intact despite the known verb-related impairments (Aveni et al., 2023). On the other hand, mounting evidence indicates challenges in verbal fluency, lexical retrieval, sentence comprehension, and pragmatic use of language, particularly in tasks requiring executive resources (Auclair-Ouellet et al., 2017; Montemurro et al., 2019). These observations have led some authors to suggest that verbal impairments in PD may not be purely linguistic but rather reflect broader executive demands, particularly in cognitively demanding situations (Smith and Caplan, 2018; Lowit et al., 2022). By contrast, non-verbal cognition, especially visuo-spatial abilities, appears to be consistently impaired in PD (Liebermann-Jordanidis et al., 2022), with deficits reported in mental rotation, spatial planning, visual attention, and visuo-constructive tasks (Liebermann-Jordanidis et al., 2022). For example, Amick et al. (2006) found that cognitively intact PD patients performed significantly more slowly and less accurately on mental rotation tests, particularly for large rotation angles (Amick et al., 2006). Other studies confirm similar difficulties with spatial planning, visual attention, and visuo-constructive tasks (e.g., complex drawings; Crucian and Okun, 2003). It is thought that these difficulties result from early dysfunctions in fronto-striatal and posterior parietal circuits (Alrazi, 2021), and may even precede overt motor symptoms in some cases (Nieto-Escamez et al., 2023).

Within the social-cognitive domain, most studies have focused on verbal ToM tasks, such as narrative-based inference or irony detection, which are often impaired in patients with PD-MCI (i.e., a transitional stage between normal cognitive functioning and dementia; Aarsland et al., 2011) and cognitively intact PD subjects (Poletti et al., 2011). These tasks require linguistic comprehension, inferential reasoning, and working memory (Oberauer, 2005), making them effective in detecting overlapping cognitive decline (Maggi et al., 2022). By contrast, non-verbal ToM performance, assessed through tasks such as interpreting animated shapes or facial expressions, appears more heterogeneous in the literature (Bora et al., 2015). While studies suggest partial preservation (Romosan et al., 2019), others report impairments (Dodich et al., 2022). Methodological differences in task sensitivity, as well as the potential interference of visuo-spatial deficits, frequently observed in PD and inherent to many non-verbal ToM tasks, are likely to contribute to this inconsistency (Poletti et al., 2011; Trompeta et al., 2021).

## Study overview, aims and hypotheses

The present study examined the interaction between processing modality (verbal vs. non-verbal) and functional domain (cognition vs. ToM) in two groups of PD patients: cognitively unimpaired PD patients and PD-MCI patients, both compared with age-matched HC. Our objective was to determine whether observed areas of decline reflect domain-specific vulnerabilities, modality-specific mechanisms, or differences in task-related cognitive demands.

More specifically, we aimed to disentangle whether differences between verbal and non-verbal performance are better explained by partially distinct underlying cognitive and neural mechanisms, or by variations in executive and processing demands associated with the tasks. Our working hypotheses were:

1) Both PD and PD-MCI groups were expected to show impairments compared to HC, with more severe deficits in the PD-MCI group across all domains and modalities (Aarsland et al., 2011; Wild et al., 2013);
2) Differences between verbal and non-verbal performance would reflect both domain-specific vulnerabilities and differences in task demands, rather than a purely modality-driven dissociation. Specifically, regarding the verbal/non-verbal dimension, we expected non-verbal cognition, particularly visuo-spatial abilities, to be more compromised, as suggested by prior literature on the early involvement of posterior cortical and fronto-striatal circuits in PD (Alrazi, 2021). By contrast, verbal cognition was expected to show relative preservation in lower-demand conditions (Altmann and Troche, 2011; Diaz et al., 2016), but selective impairment in tasks requiring higher executive and inferential load (Auclair-Ouellet et al., 2017; Montemurro et al., 2019). Within this framework, differences between verbal and non-verbal performance have been interpreted as arising from the interaction between domain-specific neural vulnerabilities and task-related cognitive demands, particularly executive control processes (e.g., working memory, inhibition, and cognitive flexibility) and inferential processes (e.g., integration of contextual information and mental state attribution), rather than reflecting a purely modality-driven dissociation (Alrazi, 2021; Liebermann-Jordanidis et al., 2022);
3) With respect to the cognition/ToM dimension, we expected the verbal component to be primarily impaired in PD-MCI, whereas the non-verbal one to be relatively preserved. Nonetheless, given the contribution of executive resources to mentalizing, we also considered the possibility of broader ToM impairments in PD-MCI in tasks requiring higher inferential demands (Bodden et al., 2010; Poletti et al., 2011; Bora et al., 2015; Rossetto et al., 2018).

To test these three hypotheses, we administered a neuropsychological battery considering all four components—verbal cognition, non-verbal cognition, verbal ToM, and non-verbal ToM—and conducted comparative analyses across the three groups.

## Methods

### Participants

Seventy-seven participants took part in the cross-sectional study (mean age = 69.81, SD = 7.20), divided into three parallel groups:

- HC: *n* = 26 (mean age = 70.81, SD = 6.50)
- PD: *n* = 28 (mean age = 69.15, SD = 7.69)
- PD-MCI: *n* = 23 (mean age = 69.29, SD = 7.51)

Participants were recruited at the IRCCS Fondazione Mondino (Pavia, Italy), either during hospitalization or through outpatient services. They provided written informed consent in accordance with ethical standards.

An *a priori* power analysis was conducted using G*Power 3.1 (Faul et al., 2009) to estimate the minimum required sample size for the multinomial logistic regression model. Assuming an odds ratio (OR) of 1.5, *α* = 0.05, power (1 − *β*) = 0.80, Pr(Y = 1|X = 1) = 0.2, 9 predictors (7 main effects, 2 interactions), and R^2^ other X = 0.1, the minimum sample was estimated at 76, confirming adequacy of the current sample (*N* = 77).

Inclusion criteria for all participants included:

- Age ≥ 50 years, selected as a cut-off to ensure consistency with the PD-specific literature and to obtain a more clinically homogeneous sample, as onset before this threshold is typically considered a distinct subgroup (early-onset PD) with specific clinical and progression features (Mehanna et al., 2022; Cilia et al., 2015)
- Education ≥ 5 years

Exclusion criteria were:

- History of psychiatric or neurological disorders, substance abuse, or uncorrected sensory deficits
- Pre-existing conditions affecting cognition (e.g., aphasia, neglect)

PD was diagnosed according to the MDS clinical diagnostic criteria for Parkinson’s disease (Postuma et al., 2015), PD-MCI was diagnosed according to the MDS Task Force criteria (Litvan et al., 2012). Global cognition was screened using the Mini-Mental State Examination (MMSE; Folstein et al., 1975).

### Materials

Neuropsychological measures were selected and organized along the four key dimensions of a 2 × 2 factorial design, with functional domain (cognition vs. ToM) and processing modality (verbal vs. non-verbal), using widely adopted and validated measures in PD research.

This approach was theory-driven and aimed at capturing broader functional domains aligned with our framework. More in detail, within each composite dimension, tasks were selected based on their conceptual coherence. Verbal cognition was assessed through measures that require lexical retrieval, semantic access, and executive control processes such as initiation and strategic search (e.g., verbal fluency, FAB). In contrast, non-verbal cognition was assessed using tasks that rely more heavily on visuospatial processing, visual attention, and cognitive flexibility (e.g., TMT), particularly in conditions requiring set-shifting and visuo-motor integration. Similarly, verbal ToM tasks (Strange Stories) rely on narrative comprehension and inferential reasoning, while non-verbal ToM tasks (Animation Task) assess the attribution of mental states based on dynamic visual information.

#### Verbal domain

##### Cognition

- Frontal Assessment Battery (FAB; Appollonio et al., 2005): assesses executive functions including inhibition, mental flexibility, and planning. It comprises six subtests scored 0–3, yielding a total range of 0–18 (higher scores indicate better executive functioning). The authors did not report internal consistency (*α*), though test–retest and inter-rater reliability were acceptable (Appollonio et al., 2005).
- Verbal fluency:
- *Phonemic fluency* (FAS; Novelli et al., 1986): generate words beginning with F, A, and S in 1 min each.
- *Semantic fluency* (Novelli et al., 1986): generate category-specific words (e.g., animals) in 1 min. Both the tests are expressed as the number of correct words generated in 1 min, with normative data suggesting averages of approximately 10–15 words per letter (phonemic FAS) and 18–25 words per category (semantic fluency) in healthy adults. They demonstrated high test–retest reliability and interrater reliability (≈ 0.90), with strong correlations between letter sets (r = 0.85–0.94; Wigdorowitz et al., 2021).

##### ToM

- Strange Stories Test (White et al., 2009): assesses higher-order mentalizing through narratives involving beliefs, irony, deception. Participant’s answers are scored as 0 (fail), 1 (partially correct), or 2 (fully correct). In the present study, eight Theory of Mind stories were administered, including scenarios involving *deception, irony, white lies, and misinterpretation of beliefs and intentions*, for a total range of 0–16.

#### Non-verbal domain

##### Cognition

- Trail Making Test (TMT; Reitan, 1956):
- *Part A* evaluates visual attention and processing speed.
- *Part B* assesses cognitive flexibility and visuo-motor integration.
- It is scored based on completion time in seconds for Parts A and B, with lower times indicating better performance. Normative cutoffs vary by age and education. It showed test–retest reliability ranging from *r* = 0.15–0.70 for Part A and *r* = 0.33–0.80 for Part B, while other sources report higher reliability (*r* = 0.76–0.94; Bracken et al., 2019).

##### ToM

- Animation Task (Castelli et al., 2000): participants watch short videos of animated geometric shapes and describe interactions. Mentalizing is scored based on intentionality attribution and response appropriateness. Control trials assess action-description without mental state inference. In the present study, participants viewed six experimental animations, preceded by two practice clips, in line with the original paradigm. It uses an intentionality scoring system with a maximum of 20 points. Normative data report a mean of 15.8 (SD = 1.5) in healthy adults. It exhibited high interrater reliability (Krippendorff’s α = 0.89), with additional interrater agreement estimations between 65% and over 90%, and acceptable ICC values for key variables (Shahrivar et al., 2020).

Executive dysfunctions were not directly assessed as an independent construct; however, tasks with high cognitive load were included to capture potential effects of executive demands on performance (e.g., FAB, TMT-B).

Therefore, any interpretation regarding its role should be considered exploratory, and grounded in prior literature linking executive control with mentalizing and complex reasoning (Colman et al., 2011; Foley et al., 2019; Lowit et al., 2022).

### Procedure

All the assessments were conducted individually in a quiet room at the IRCCS Mondino Institute. Tests were administered in paper-and-pencil format, except for the Animation Task, which was presented via computer: here participants responded orally and their answers were later transcribed. The testing session included standardized assessments of global cognition, verbal and non-verbal cognitive performance, and ToM. Trained experimenters administered all tasks using standardized instructions.

The present study has been reviewed and approved by the Ethics Committee of the S. Matteo Hospital, Pavia, Italy. The participants provided their written informed consent to participate, in accordance with the Declaration of Helsinki.

### Data planning

With regard to the data cleaning process, missing data were addressed using group mean imputation, as described in Allison (Allison, 2003). This approach involved considering each variable individually before aggregating them into clusters. Cognitive measures were expressed as Equivalent Scores (0–4) based on Italian normative data, whereas ToM measures were analyzed using raw scores due to the lack of standardized normative references. For inferential analyses, all variables were standardized (z-scores) to ensure comparability across measures with different scales (Brooks et al., 2009).

Outliers were treated using the interquartile range (IQR) method, leveraging the functionality of the R package *dplyr* (Wickham et al., 2023) to calculate Q1 and Q3 values for each measure and define the acceptable range as [Q1–1.5 × IQR, Q3 + 1.5 × IQR], as in Kafadar and colleagues (Kafadar et al., 1989).

In order to reach our main aims, we verified the assumptions for the ANOVA, running the Shapiro–Wilk test to check for normality of residuals, and the Levene Test to check for the homogeneity of variances on every measure included in our analysis. Results from the Shapiro–Wilk and Levene tests revealed that the assumptions for ANOVA were not respected for some of our measures (i.e., Levene *p* < 0.001 for non-verbal cognition).

In light of this, we used the non-parametric Kruskal-Wallis test to obtain more robust results (McKight and Najab, 2010). Here the group was the independent variable, with the aforementioned measures representing the dependent variables. We ran the Dunn test as a post-hoc test, applying the Bonferroni correction, as described in Dinno (Dinno, 2015). Effect sizes (η^2^) were computed for Kruskal-Wallis tests to quantify the magnitude of group differences.

As a second analysis, we ran a multinomial logistic regression analysis, with the three-level “group” variable as a dependent one, to examine the relative contribution of verbal and non-verbal cognitive and ToM measures in discriminating between cognitively normal PD, PD-MCI, and HC. As main predictors we inserted all the verbal measures (i.e., verbal cognition and verbal ToM) and all the non-verbal measures (i.e., non-verbal cognition and non-verbal ToM). We set PD-MCI as the reference category. Thus, the odds ratios reported for the comparison between HC and PD-MCI indicate the likelihood of belonging to the HC group relative to PD-MCI for higher predictor values, whereas those reported for the comparison between PD and PD-MCI indicate the likelihood of belonging to the PD group relative to PD-MCI.

## Results

### Descriptive statistics

The three groups were found to be equivalent in terms of size, age, schooling, and percentage of females (See Table 1 for complete descriptive statistics for each group: raw scores—or Equivalent scores, for cognitive tests—are reported).

**Table 1:** Descriptive statistics.

Group	N	Years of education (mean)	Female (%)	MMSE ± SD
HC	26	9.19	46	**29.00** ± 1.34
PD	28	10.62	50	27.78° ± 1.98
PD-MCI	23	10.53	57	27.74 ± 1.97
Total	77	10.08	51	28.16 ± 1.87
*p* value§	0.781	0.379	0.766	< 0.001

§Kruskal-Wallis test. HC, healthy controls; PD, patients with Parkinson’s disease and normal cognition; PD-MCI, patients with Parkinson’s disease and MCI. *Dunn’s test with Bonferroni correction revealed significant differences between the HC and PD-MCI groups (*p* < 0.001; bold values in the table) and between the PD and PD-MCI groups (*p* < 0.025; values marked with °), with positive z values indicating better performance in the former groups; since Dunn’s test relies on rank distributions rather than on group means, these results capture distributional differences across groups.

### Main analyses

The means and standard deviations for every variable of interest are illustrated in Table 2 (Raw descriptive statistics for each individual task before aggregation are reported in Supplementary Table S1).

**Table 2:** Means and standard deviations for every measure of interest as a function of group.

Group	Processing modality	Functional domain	Min	Max	Range	M ± SD
HC	Verbal	Cognition	2.00	4.00	2.00	3.55 ± 0.59
		ToM	4.00	8.00	4.00	6.27 ± 1.00
	Non-verbal	Cognition	0.50	4.00	3.50	3.54 ± 1.92
		ToM	9.00	23.00	14.00	14.62 ± 3.70
PD	Verbal	Cognition	1.67	4.00	2.33	2.76 ± 0.95
		ToM	0.00	8.00	8.00	5.00 ± 1.97
	Non-verbal	Cognition	0.50	4.00	3.50	2.71 ± 1.40
		ToM	8.00	22.00	14.00	14.75 ± 3.52
PD-MCI	Verbal	Cognition	0.33	4.00	3.67	2.73 ± 0.95
		ToM	2.00	8.00	6.00	4.94 ± 1.95
	Non-verbal	Cognition	0.00	4.00	4.00	2.59 ± 1.40
		ToM	8.00	24.00	16.00	14.51 ± 3.58

HC, healthy controls; PD = patients with Parkinson’s disease and normal cognition; PD-MCI, patients with Parkinson’s disease and mild cognitive impairment. Verbal Cognition and Non-verbal Cognition are expressed as Equivalent Scores (0–4) derived from Italian normative data for FAB, phonemic/semantic fluency, and TMT-A/B. Strange Stories scores represent raw performance on the mentalistic stories only (range 0–8), in line with standard ToM scoring procedures; physical-control stories were scored separately and not included in the ToM measure. Triangles-ToM scores represent non-verbal ToM performance and were calculated as the sum of intentionality and appropriateness ratings for the mentalizing animations (T clips; range 8–24). Control animations (A clips) were excluded from the ToM index. No normative scoring system exists for Strange Stories or for the Animation Triangles Task; therefore, ToM measures are here reported using raw scores. All inferential analyses were conducted on internally standardized scores (z-scores), computed within the full sample to allow comparability across measures.

Table 3 illustrates the correlations between measures.

**Table 3:** Correlation matrix.

	Verbal cognition	Verbal ToM	Non-verbal cognition	Non-verbal ToM
Verbal cognition	–	**0.35**	**0.43**	0.067
Verbal ToM		–	0.196	0.007
Non-verbal cognition			–	**0.24**

Spearman’s rank-order correlation matrix among the four key measures. Significant correlations are reported in bold.

As regards between-group comparisons across verbal and non-verbal cognition and ToM, significant differences emerged for several, but not all, variables.

In the verbal domain, cognitive performance differed significantly across all three groups (HC, PD, PD-MCI; χ^2^ = 27.46, *p* < 0.001, η^2^ = 0.34), with healthy controls outperforming PD, and PD outperforming PD-MCI.

For verbal ToM, HC showed better performance than PD-MCI (χ^2^ = 9.07, *p* = 0.01, η^2^ = 0.10).

In the non-verbal domain, cognitive performance was significantly lower in PD-MCI compared to both HC and PD (χ^2^ = 16.17, *p* < 0.001, η^2^ = 0.19), while no significant group differences were observed for non-verbal ToM (χ^2^ = 0.11, *p* = 0.33, η^2^ = 0.00; Table 4).

**Table 4:** Kruskal-Wallis and Dunn results.

Processing modality	Functional domain	χ^2^	df	η^2^	*p*-value	Post-hoc (Significant Comparisons)
Verbal	Cognition	27.46	2	0.34	**<0.001**	**2–3**
					**<0.001**	**2–4**
					**<0.001**	**3–4**
	ToM	9.07	2	0.10	**0.01**	**2–4**
Non-verbal	Cognition	16.17	2	0.19	**<0.001**	**2–4**
						**3–4**
	ToM	0.11	2	0.00	0.33	None

Kruskal-Wallis test and Dunn Test with Bonferroni correction. For *post-hoc* comparison group codes are as follows: 2 = HC, 3 = PD, 4 = PD-MCI. HC, healthy controls; PD, patients with Parkinson’s disease and normal cognition; PD-MCI, patients with Parkinson’s disease and mild cognitive impairment. η^2^ values represent effect sizes computed for Kruskal-Wallis tests. Significant results are reported in bold.

Concerning predictors of group membership, multinomial logistic regression analyses revealed that verbal cognition was significant (*p* = 0.001), with higher scores substantially increasing the likelihood of belonging to the HC group relative to PD-MCI (OR = 8.64, 95% CI [2.39–31.27]). A similar effect was observed for the PD group (OR = 4.81, 95% CI [1.62–14.27], *p* = 0.005).

Verbal ToM did not significantly predict group membership in either comparison (*p* > 0.39).

Regarding non-verbal variables, non-verbal cognition significantly predicted group membership, with higher scores associated with increased odds of belonging to HC (OR = 2.93, 95% CI [1.33–6.46], *p* = 0.008) and PD groups (OR = 2.08, 95% CI [1.13–3.81], *p* = 0.018) relative to PD-MCI.

Non-verbal ToM did not show significant effects (*p* > 0.39).

Overall, goodness-of-fit indices indicated a strong model fit (Nagelkerke R^2^ = 0.54), suggesting that cognitive variables provide substantial explanatory power in distinguishing PD-MCI from the other groups (Table 5).

**Table 5:** Results of the multinomial logistic regression analysis.

Group comparison	Predictor	β	SE	z	*p*	OR (95% CI)
HC vs PD-MCI	Verbal cognition	2.16	0.66	3.28	**0.001**	8.64 (2.39–31.27)
	Verbal ToM	0.22	0.26	0.84	0.399	1.24 (0.75–2.06)
	Non-verbal cognition	1.07	0.40	2.66	**0.008**	2.93 (1.33–6.46)
	Non-verbal ToM	−0.10	0.12	−0.84	0.399	0.90 (0.72–1.14)
PD vs PD-MCI	Verbal cognition	1.57	0.56	2.83	**0.005**	4.81 (1.62–14.27)
	Verbal ToM	−0.15	0.21	−0.74	0.462	0.86 (0.57–1.29)
	Non-verbal cognition	0.73	0.31	2.37	**0.018**	2.08 (1.13–3.81)
	Non-verbal ToM	−0.10	0.11	−0.85	0.396	0.91 (0.73–1.13)

Multinomial logistic regression analysis. Reference category = PD-MCI group. Odds ratios (OR > 1) indicate increased odds of belonging to the comparison group (HC or PD) relative to PD-MCI for higher predictor values. Significance results are written in bold. HC, healthy controls; PD, Parkinson’s disease with normal cognition; PD-MCI, Parkinson’s disease with mild cognitive impairment. Significant results are reported in bold.

## Discussion

The present study aimed to clarify how cognitive and social-cognitive impairments in PD unfold across two critical modalities, verbal and non-verbal, within the cognitive and ToM domains.

Consistently with existing literature (Wild et al., 2013; Menozzi et al., 2025) and with our first hypothesis, between-groups comparison analyses revealed that both PD and PD-MCI performed worst compared to HC, with the PD-MCI group exhibiting the most severe impairments across cognitive and social-cognitive domains. Importantly, the observed decline was not uniform, with differential vulnerability in both domains and modalities, suggesting that task-specific cognitive demands modulate performance. This is aligned with the notion that tasks with higher cognitive load may exhaust executive resources such as working memory, set-shifting, particularly challenging individuals with reduced cognitive resources (Ravizza et al., 2012), even if it has to be specified that executive functions were not directly modeled and their role can be inferred from task demands and performance patterns (Colman et al., 2011; Lowit et al., 2022).

As expected, non-verbal cognition emerged as more compromised, primarily in tasks tapping visuo-spatial processing (Amick et al., 2006; Liebermann-Jordanidis et al., 2022). This was reflected by significantly poorer TMT performance in the PD-MCI group than both PD and HC groups. The difference was evident for both parts of the test, but particularly pronounced in TMT-B: indeed, PD-MCI showed the worst performance in this part, placing higher demands on cognitive flexibility. The overall index confirmed this pattern, in accordance with previous evidence linking early visuo-spatial deficits in PD to dysfunctions in fronto-striatal and posterior parietal networks, areas that are crucial for spatial attention, planning, and visuo-motor integration (Alrazi, 2021; Liebermann-Jordanidis et al., 2022).

Although verbal cognition was affected, it showed a comparatively milder decline. Specifically, scores for phonemic and semantic fluency were significantly lower in PD-MCI participants than in HC, but there were minimal differences between PD and PD-MCI participants. PD-MCI participants also achieved lower scores on the FAB, indicating difficulties with tasks involving inhibition, flexibility, and planning—functions often linked to executive control (Auclair-Ouellet et al., 2017; Montemurro et al., 2019),^1^ which is required in verbal performance (Fedorenko et al., 2012; Novakova et al., 2023). This is also supported by neuroimaging evidence (Grossman et al., 2003) further underscoring the interplay between verbal processing and executive resources in PD, highlighting the recruitment of frontal systems under higher task demands. Taken together, these findings provide a more nuanced view of the preservation of verbal functions in PD, challenging the perception of uniformity and suggesting a more differentiated picture (Altmann and Troche, 2011; Diaz et al., 2016).

Turning to the social-cognitive axis, verbal ToM was selectively impaired in PD-MCI, whereas non-verbal ToM did not show significant group differences and appeared relatively preserved. This apparent preservation should be interpreted with caution, as it may reflect limited task sensitivity rather than a true sparing of non-verbal ToM abilities, although it is also broadly consistent with previous findings (Bora et al., 2015), suggesting that such tasks may be more sensitive to cognitive deterioration. On the other hand, lower verbal ToM scores were observed in individuals with concurrent executive deficits (Seyfarth and Cheney, 2015; Rossetto et al., 2018; Montemurro et al., 2019; Rosch, 2024).

It is important to acknowledge that several approaches, including in the aging literature, conceptualize ToM as comprising partially distinct cognitive (i.e., inference of beliefs and intentions) and affective (i.e., understanding of emotions) components (Bottiroli et al., 2016; Zhou et al., 2022; Merlini et al., 2026). While this distinction provides a useful interpretative framework, it has not directly been addressed in the present study, which instead decided to focus on a different distinction, also in light of the fact that the tasks employed primarily tap into the cognitive component of ToM, with the Strange Stories Test relying on narrative comprehension and inferential reasoning about others’ beliefs and intentions, rather than on affective mental state attribution. The very fact that differentiated patterns emerge even within the cognitive component alone seems to suggests that these processes, although partially dissociable, can still be conceptualized within a unified model.

Regression analyses further underscored the novelty of these findings: neither verbal nor non-verbal ToM significantly predicted group classification, whereas both verbal and non-verbal cognitive measures were strong predictors, emphasizing the superior discriminative power of core cognitive variables compared to ToM measures in distinguishing stages of cognitive decline in PD.

This study presents several limitations that should be acknowledged. First, although the 2 × 2 design offered a structured comparison between modalities and domains, the neuropsychological battery was necessarily constrained in scope. The verbal and non-verbal tasks, while representative, may not fully capture the complexity of these constructs: future studies could benefit from integrating more targeted or context-sensitive measures to complement current approaches, particularly in the social-cognitive domain. Furthermore, the use of composite indices, while theoretically motivated, may have masked task-specific dissociations: it may be of interest to disentangle these components using more targeted analyses. The cross-sectional nature of the study limits inference on trajectories of modality-specific decline: longitudinal research may be needed to clarify whether the observed dissociations persist, converge, or diverge over time. Then, while the inclusion of both PD and PD-MCI groups allowed for a fine-grained comparison, the sample size—although adequately powered for our analyses—might limit the generalizability of subgroup-specific conclusions. Moreover, in future works it would be useful to consider the association between symptomatology and verbal/non-verbal tasks. A further limitation concerns the assessment of non-verbal ToM: the absence of group differences in our study may partially reflect reduced task sensitivity or the heterogeneity of the neural substrates underlying this function, as suggested in previous work (Molenberghs et al., 2016). Another aspect to be considered concerns the lack of assessment of neuropsychiatric symptoms such as depression and apathy, which are known to influence cognitive performance in PD (Giannouli and Tsolaki, 2021). These factors may affect executive functioning, motivation, and task engagement, especially in tasks with higher cognitive and inferential demands, and therefore cannot be excluded as contributing to the present findings: as a future development, it may be interesting to include these variables to better clarify their role in cognitive and social-cognitive performance. To conclude, no clinical data were considered in this study. However, all patients were recruited on the basis of a diagnosis made by neurologists in a controlled clinical setting.

Despite these constraints, the study contributes novel insights into selective cognitive and social-cognitive deficits in PD, widely recognized as a multifactorial disorder, affecting a broad range of cognitive and functional systems (Kaur et al., 2019). Our findings highlight dissociations that are critical for understanding disease progression and cognitive architecture. Differentiated vulnerabilities emerge across domains, with modality acting as a modulatory factor, particularly in interaction with task complexity and executive demand (Kehagia et al., 2013; Jaywant et al., 2014; Bora et al., 2015; Montemurro et al., 2019; Alrazi, 2021; Gonzalez-Latapi et al., 2021).

## Data Availability

The datasets presented in this study can be found in online repositories. The names of the repository/repositories and accession number(s) can be found below: [Zenodo; Reservation: 10.5281/zenodo.20052883.

## Footnotes

1 It is possible to refer to supplementary materials for the detail of results in every task before being aggregated.
