# Supplementary material for: Domain-driven verbal and non-verbal dissociations in cognition and social cognition in Parkinson’s disease
Source: Front Psychol. 2026 May 21;17:1775416. doi: 10.3389/fpsyg.2026.1775416 (PMC13233367; doi:10.3389/fpsyg.2026.1775416)
Supplement: Supplementary file 1 [file Table_1.docx]

**Supplementary material.**

Table. Descriptive statistics of raw scores for every measure of interest before being aggregated

| Task | Group | Mean | SD |
| --- | --- | --- | --- |
| TMT-A | HC | 57.73 | 26.56 |
|  | PD | 65.67 | 29.29 |
|  | PD-MCI | 106.57 | 62.59 |
| TMT-B | HC | 137.65 | 77.62 |
|  | PD | 111.29 | 50.50 |
|  | PD-MCI | 245.83 | 134.86 |
| FAB | HC | 17.0 | 1.12 |
|  | PD | 15.64 | 2.02 |
|  | PD-MCI | 13.25 | 2.00 |
| Phonemic fluency | HC | 35.22 | 10.28 |
|  | PD | 34.9 | 9.58 |
|  | PD-MCI | 27.63 | 9.69 |
| Semantic fluency | HC | 43.12 | 6.91 |
|  | PD | 39.00 | 6.93 |
|  | PD-MCI | 34.11 | 8.87 |
| Verbal ToM (Strange Stories) | HC | 6.15 | 1.06 |
|  | PD | 5.09 | 1.47 |
|  | PD-MCI | 4.91 | 1.5 |
| Non-verbal ToM (Triangles) | HC | 6.41 | 1.06 |
|  | PD | 6.01 | 1.46 |
|  | PD-MCI | 6.17 | 1.20 |
